# Signs and signals limiting myocardial damage using PICSO: a scoping review decoding paradigm shifts toward a new encounter

**DOI:** 10.3389/fcvm.2023.1030842

**Published:** 2023-05-09

**Authors:** Werner Mohl, Zlata Kiseleva, Alem Jusic, Matthäus Bruckner, Robert M. Mader

**Affiliations:** ^1^ Department of Cardiac Surgery, Medical University of Vienna, Vienna, Austria; ^2^ Department of Medicine I, Comprehensive Cancer Center of the Medical University of Vienna, Vienna, Austria

**Keywords:** acute coronary syndromes, acute heart failure, PICSO, molecular signaling, non-coding RNA, hypothesis, myocardial development and regeneration

## Abstract

**Background:**

Inducing recovery in myocardial ischemia is limited to a timely reopening of infarct vessels and clearing the cardiac microcirculation, but additional molecular factors may impact recovery.

**Objective:**

In this scoping review, we identify the paradigm shifts decoding the branching points of experimental and clinical evidence of pressure-controlled intermittent coronary sinus occlusion (PICSO), focusing on myocardial salvage and molecular implications on infarct healing and repair.

**Design:**

The reporting of evidence was structured chronologically, describing the evolution of the concept from mainstream research to core findings dictating a paradigm change. All data reported in this scoping review are based on published data, but new evaluations are also included.

**Results:**

Previous findings relate hemodynamic PICSO effects clearing reperfused microcirculation to myocardial salvage. The activation of venous endothelium opened a new avenue for understanding PICSO. A flow-sensitive signaling molecule, miR-145-5p, showed a five-fold increase in porcine myocardium subjected to PICSO.

Verifying our theory of “embryonic recall,” an upregulation of miR-19b and miR-101 significantly correlates to the time of pressure increase in cardiac veins during PICSO (*r*^2^ = 0.90, *p* < 0.05; *r*^2 ^= 0.98, *p* < 0.03), suggesting a flow- and pressure-dependent secretion of signaling molecules into the coronary circulation. Furthermore, cardiomyocyte proliferation by miR-19b and the protective role of miR-101 against remodeling show another potential interaction of PICSO in myocardial healing.

**Conclusion:**

Molecular signaling during PICSO may contribute to retroperfusion toward deprived myocardium and clearing the reperfused cardiac microcirculation. A burst of specific miRNA reiterating embryonic molecular pathways may play a role in targeting myocardial jeopardy and will be an essential therapeutic contribution in limiting infarcts in recovering patients.

## GRAPHICAL ABSTRACT

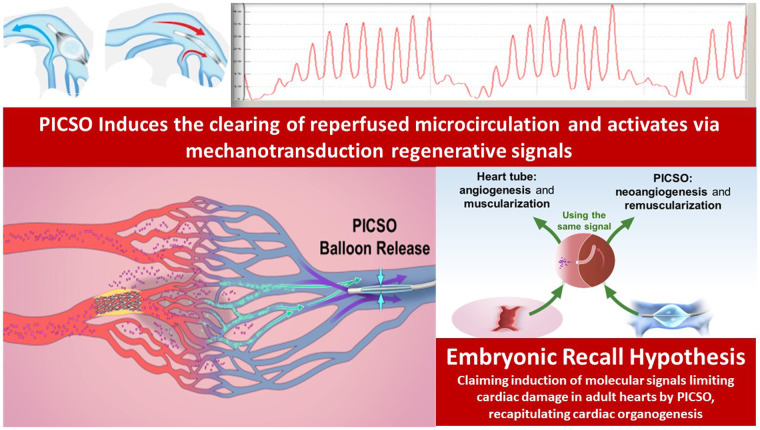

## Introduction

Nothing conserves ischemic myocardium better than early reperfusion. However, an uncontrolled inflow of oxygenated blood into the damaged microcirculation has many side effects, a phenomenon known to cardiac surgeons since the pre-cardioplegia era in open-heart surgery (1–3).

One option for myocardial protection evolved from an old idea when Pratt used cardiac veins to access the myocardium in isolated feline hearts (4). This concept reached a climax in Beck's procedure, reversing coronary flow in cardiac veins in patients with severe atherosclerosis, a staged surgical procedure connecting the aorta and the coronary sinus and in a second step narrowing the outflow (5). Although rapidly abandoned, this concept became popular again in the 1980s as an interventional method (6).

We modified the concept of arterial retroperfusion after a critical review of forces developed in the coronary circulation and developed pressure-controlled intermittent coronary sinus occlusion (PICSO) (7). After a successful experimental testing, we conducted our first clinical observation in a small, randomized trial during the reperfusion period in coronary artery bypass grafting (CABG) patients. The surgical application back then, starting PICSO 5 min after the onset of reperfusion, mirrors the situation today in interventional reperfusion in acute coronary syndromes (ACS). We concluded that PICSO during early reperfusion is safe and effective in conserving myocardial region dysfunction and stated: “Parallel to these evaluations, our first and foremost research efforts should be directed toward applying PICSO to the setting of acute myocardial infarction. Whether we can fully establish a benefit in this particular setting will be decisive for a potentially widespread clinical acceptance of the technique*”* (8).

It took exactly 30 years after this publication until another randomized trial started, recruiting patients with acute coronary syndromes investigating PICSO in a prospective, multicenter, randomized, controlled study during reperfusion aiming to reduce infarct size. Currently, 10 PICSO trials are listed on *clinicaltrials.gov*; five are completed, one is terminated, one is not yet recruiting, and recently two, among one the randomized trial, are recruiting. The objective of the randomized trials is “*to assess the efficacy and safety of Pressure-controlled intermittent Coronary Sinus Occlusion (PICSO) therapy started post flow restoration but before stenting during the percutaneous coronary intervention (PCI) compared to standard PCI in the setting of acute ST-segment elevation anterior myocardial infarction (STEMI)*” (ClinicalTrials.gov: NCT03625869, NCT04958421).

Current clinical trials of PICSO in early reperfusion mimic the study objective of our first trial in open-heart surgery and represent the mainstream of PICSO research.

In this scoping review, we show that the mainstream PICSO research on infarct size limitation and myocardial washout clearing microcirculation is interrupted by a definition of counterintuitive findings dictating paradigm changes in research, broadening the concept of tissue regeneration. Although not well understood at the time of publication, the washout capacity of PICSO^,^ as verified by Kenner in 1985, using blood density measurements during controlled sequences of coronary sinus occlusions, was the first indication of a branching point. The underappreciated complexity of Kenner's microhematocrit measurements in cardiac veins is now, in retrospect, the critical, still valid crossroad of PICSO research. Although corroborated by several authors using different methodologies of coronary venous retroperfusion and measurement modalities, like contrast disappearance or xenon washout, the impact and consequence of this scientific achievement, not well understood, remained unscrutinized (9–14).

Over the years, PICSO research was the target of only a few. However, exciting data besides reducing myocardial jeopardy have been collected over time and are available in the literature and give a chronological order for the scoping review presented here (15).

The focus of mainstream PICSO research was myocardial salvage.

**Figure 1:**
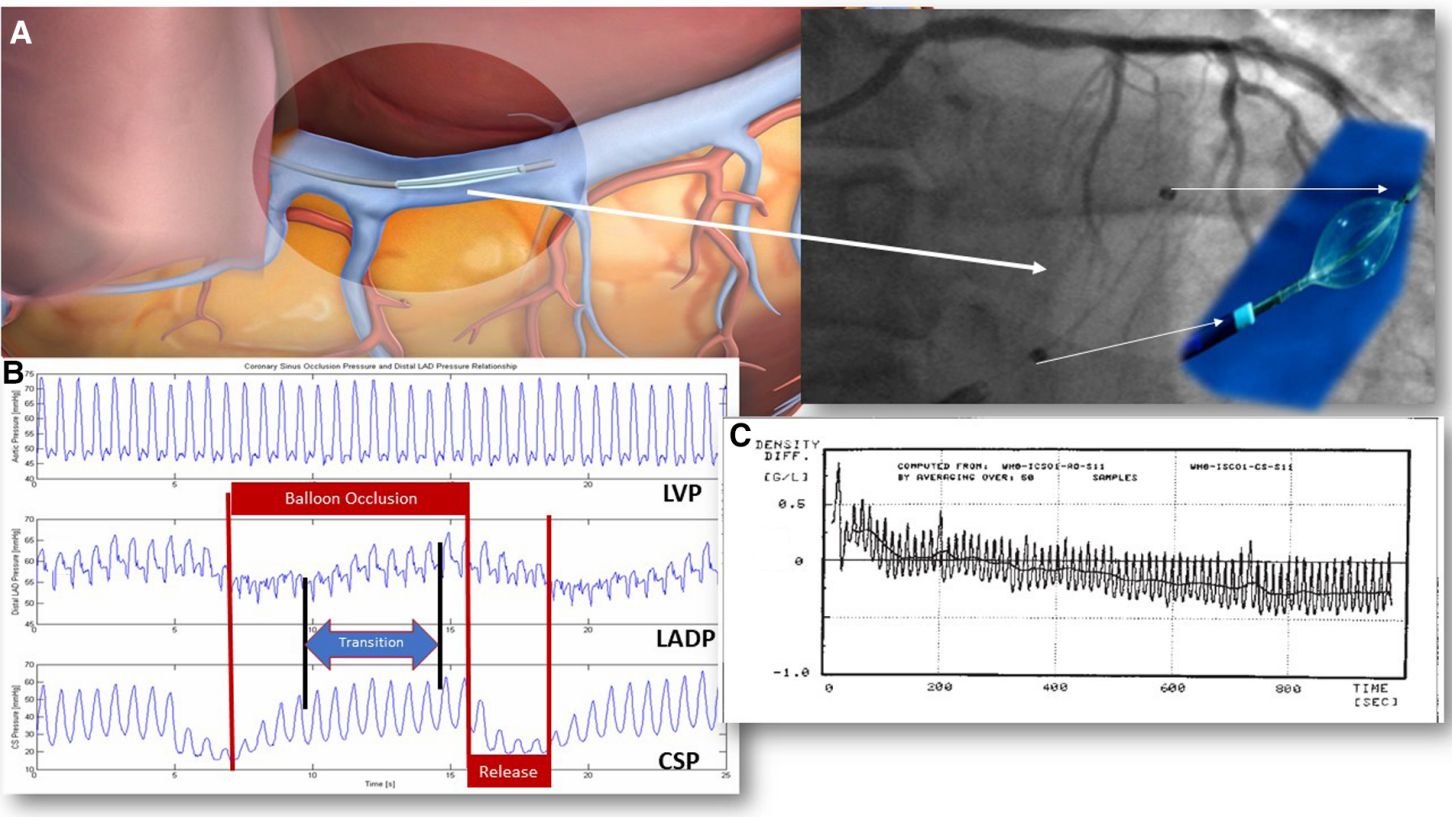
PICSO mechanisms in acute coronary syndromes. PICSO therapy duration tested clinically varies from 20 to 40 min. In experiments, PICSO for up to 6 h was studied. Optimal PICSO duration in ACS is considerably longer than in patients with heart failure due to the ongoing reperfusion injury and early pressure and flow changes in reperfused myocardium. (**A**) The position of the coronary sinus: 1. Note the position of the catheter in relation to the unobstructed venous drainage of the posterior cardiac vein in a schematic of the PICSO catheter in the coronary sinus and its fluoroscopic image. (**B**) Coronary sinus pressure during PICSO: 1. LVP is unaffected by PICSO. 2. The pressure in the occluded coronary artery (LADP) cycles according to the pressure increase in the coronary sinus (CSP). Note the transition time between CSP and LADP. Optimal access to deprived zones depends on optimal retroperfusion reaching a plateau in pressures monitored in the occluded coronary artery (LADP). Cycling of pressure sequences defines the amount of redistribution of flow and washout. The quality of the PICSO therapy is related to this pressure plateau since it depicts the amount of retroperfusate in the deprived myocardium. The blue arrow symbolizes the transition time the blood needs to enter the deprived microcirculation from cardiac veins. 3. The pressure in the coronary sinus (CSP) is an input signal for the feedback loop limiting the balloon occlusion time. Red arrows symbolize the balloon occlusion and release cycles. (**C**) Pressure tracing of blood density during experimental PICSO. Note the decline in blood density in the coronary sinus compared to the unchanged density in the aorta over a period of approximately 20 min assessing the microhematocrit showing a net washout. Each peak is one PICSO cycle. See Moser and Kenner's “The arteriovenous blood density gradient as an index for myocardial function 1988/2002” (69). LVP, left ventricular pressure; PICSO, pressure-controlled intermittent coronary sinus occlusion; ACS, acute coronary syndrome.

Table 1 depicts a description of mainstream PICSO research and paradigm changes. Figure 1 illustrates the PICSO principles. Myocardial salvage was the focus of early PICSO research and was first formulated in the pre-revascularization period. However, unexpected experimental and clinical findings followed and suggested additional beneficial molecular effects besides salvage, representing another essential branching point warranting a focus change (16). The first step toward a paradigm shift became necessary when evaluating long-term results using lysis therapy with and without PICSO in a historical Japanese study first described by Komamura in 1989, showing a 30% reduction of infarct size (16, 17). In 2008, we re-analyzed the long-term outcome of the patients studied by Komamura in 1980s. The series of patients treated with PICSO in Japan showed an impressive risk reduction in restenosis and major adverse cardiac events (MACE), outnumbering the 30% reduction of infarct size (16).

**Table 1:** The PICSO research in context.

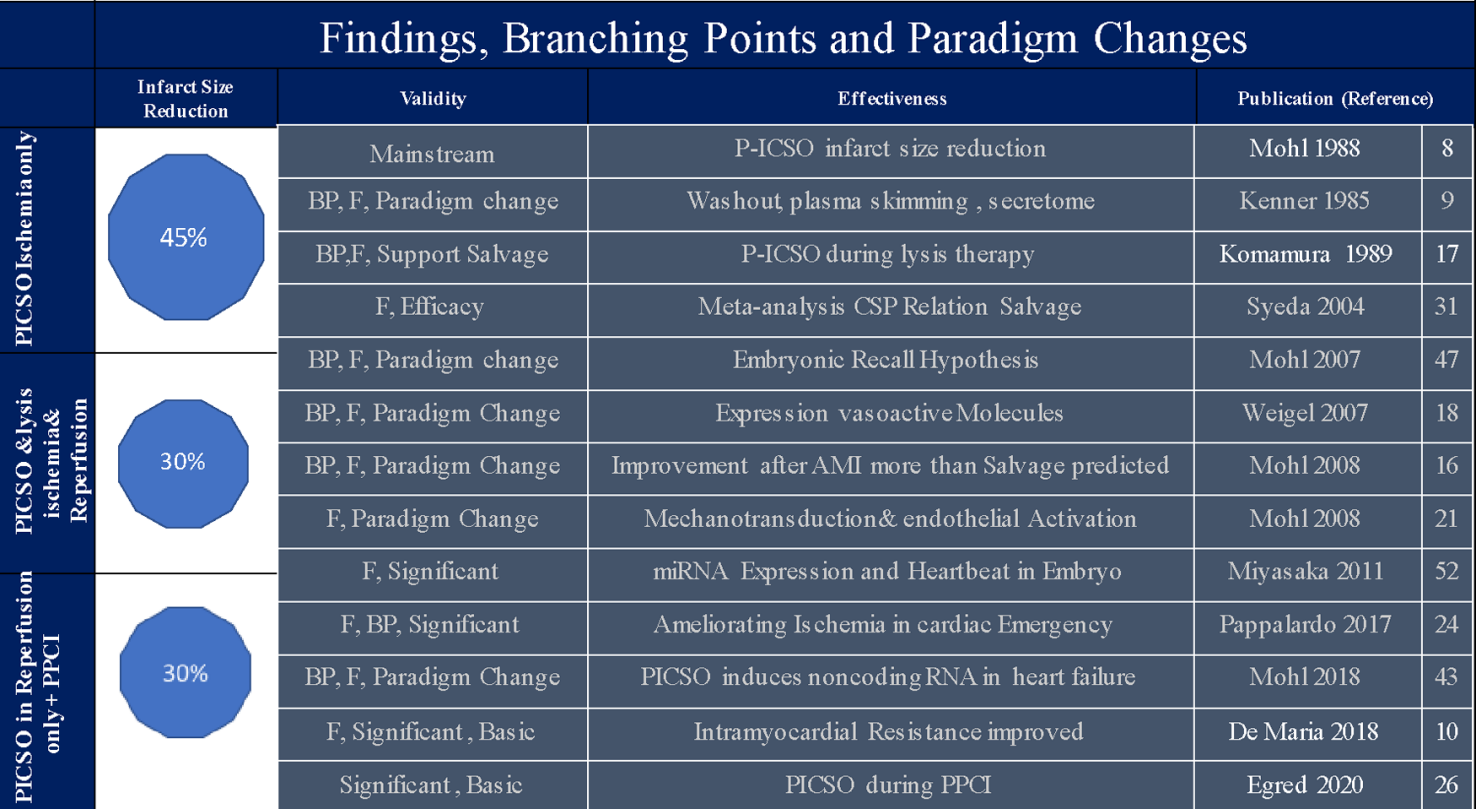

PICSO, pressure-controlled intermittent coronary sinus occlusion; BP, branching point; F, first essential publication.

The mainstream of PICSO myocardial salvage is on the left, and the amount of salvage depends on the concomitant revascularization strategy. On the other hand, scientific evidence dictating paradigm changes are arrows on the right leading the PICSO perspective from myocardial salvage to tissue regeneration. PICSO and infarct size reduction references, authors and year of publication in white: ischemia only 1984; ischemia and reperfusion (lysis) 1989; during reperfusion only 2018 and 2020.

We concluded that the prevention of MACE and restenosis of the culprit lesion was higher than salvage predicted, indicating that additional PICSO effects were present. Therefore, clearing debris after embolization during clot lysis and preventing subsequent inflammatory responses may have played a role in avoiding restenosis and MACE (16).

In agreement with these clinical observations are additional molecular consequences in porcine hearts subjected to experimental myocardial infarction and PICSO complementing this narrative (18). Reinitiating scientific interest, especially in the primary mechanism of PICSO, expanded the potential therapy for patients with heart failure (19–23). Consequently, a significant improvement in cardiac function in patients after open-heart surgery with acute failing hearts highlights the PICSO therapy in the most challenging situation of cardiogenic shock (24). In the report by Pappalardo et al. of two patients with post-cardiotomy cardiogenic shock, the two known effects of PICSO (redistribution of flow and washout) and the theoretically proposed regenerative molecular signaling seem to potentiate both effects of ameliorating ischemia and recovery of cardiac function and can be seen as a crucial synergistic effect. These counterintuitive results define a second branching point directing further research on acute cardiac failure.

Suppose we consider the divergencies of PICSO findings and the changing environment from the conceptualization of PICSO decades ago and today's modern technology, we must redefine our clinical targets and expectations. For example, as seen in Table 1, infarct size reduction differs when PICSO is applied without reperfusion, during or after revascularization.

As in all medical therapies, the intermittent drainage impediment in the coronary circulation as a dose–response parameter of PICSO to ameliorate ischemia must be defined. Therefore, *dosis sola facit venenum* prevails! Paracelsus, the brilliant medieval scholar and physician (1493–1541), formulated “the dose makes the poison,” which is also relevant in myocardial drainage limitation. Gradual and timed outflow resistance may be beneficial in ameliorating ischemia. However, sudden and complete occlusion of outflow of cardiac veins is detrimental and must be avoided. The experimental and later clinical evidence shows that PICSO induces salvage by redistributing flow into the deprived myocardium (25, 26). Balloon blockade throughout several cardiac cycles elevates the pressure in cardiac veins, reversing venous flow from normal perfusion territories into deprived microcirculatory zones, and can be detected in occluded coronary arteries after a transition time, which is related to the amount of the underperfused myocardium. Prolonging occlusion of cardiac veins, however, may reduce coronary inflow, as shown in a series of experiments varying occlusion release cycles limiting cardiac vein flow (27).

In this analysis, we found that optimal PICSO cycles may vary but consist of approximately 9 s of inflation, followed by rapid deflation lasting about 3.5 s (26, 27). The inflation length during a PICSO cycle depends on reaching a systolic coronary sinus pressure plateau during the occlusion phase. The concomitant increase in the occluded coronary artery is an essential parameter of the quality of retroperfusion. Therefore, the occlusion release cycles must be personalized and optimized and rely primarily on the amount of deprived myocardium, contractility, and arterial inflow through collaterals (27). Present technology uses algorithms for automated feedback control. The sudden reopening of venous blockade increases the washout of debris and toxic byproducts generated during reperfusion.

**Figure 3:**
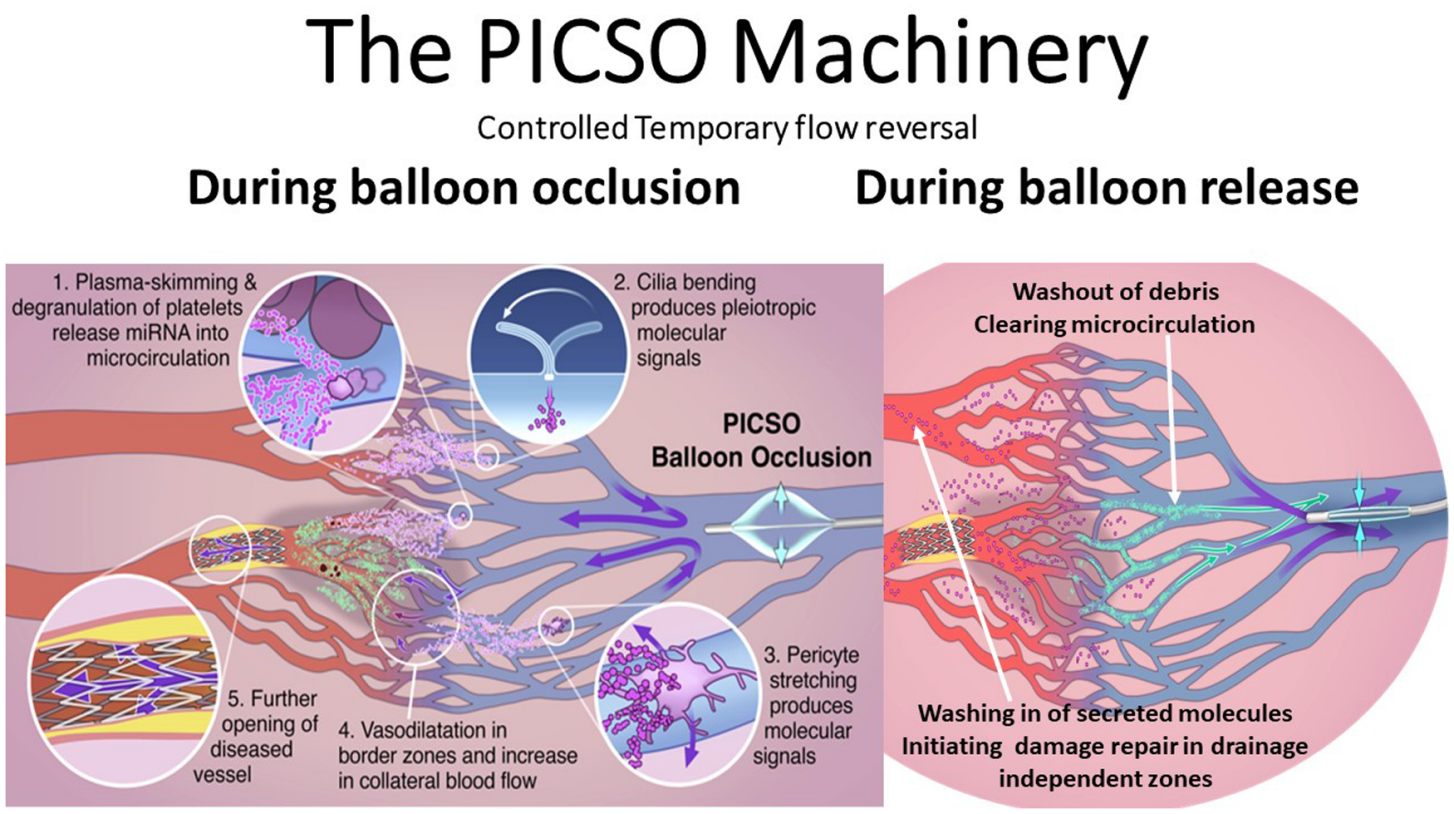
Note the dual mechanisms of the schematic sequence of events during balloon occlusion and release. First, the dose–response-dependent redistribution of venous flow during balloon occlusion and washout during balloon release creates a turnover of perfusion in the deprived zones. Second, as theorized in our “Embryonic Recall” hypothesis, the threshold-dependent activation of regenerative signaling pathways occurs. As claimed, bending intravascular cilia and stretching pericytes initiate mechanochemical feedback loops via activation of the cellular cytoskeleton. Degradation of platelets during the plasma skimming process may also play a role in releasing soluble signaling molecules. **Plasma skimming** is the central mechanism of PICSO and has been studied with blood density measurements, monitoring hematocrit changes in cardiac veins. PICSO shows microhematocrit changes with an increase during the balloon occlusion phase, shifting plasma backward into the microcirculation and leaving cellular components in more prominent cardiac veins. More extended periods of PICSO show trends of reduced density in the coronary sinus, thus indicating washout. PICSO, pressure-controlled intermittent coronary sinus occlusion.

Another deviation from the mainstream PICSO research was the observation that systolic backpressure produces a plasma skimming effect at the branching points of venous vasculature, shedding plasma into the ischemic/reperfused microcirculation (28). In addition, the degranulation of platelets and exosome secretion may be another source of signaling molecules (see Figure 3). Although unnoticed, this effect needed an alternative perception and conditioning of dealing with the PICSO therapy.

Differences in myocardial salvage are depicted in Table 1, with the most impressive salvage in nonreperfused animals. Today's clinical routine with early interventional revascularization is different and challenges the salvage potential of PICSO. In scoring infarct size reduction, differences in infarct size delineation must be considered, especially using the widely accepted technique using cardiac magnetic resonance imaging (CMR). Since coronary flow grades, pre- and post-procedure are predictors of myocardial salvage derived by CMR; combining both methods prove the significance of infarct size reduction during reperfusion by PICSO in upcoming randomized trials (10, 29). However, limitations in interpretations apply. The first scientific report in 1984 described the salvage potential of the PICSO method in animals in using planimetric methods to calculate the area at risk (AAR) and salvage following technetium perfusion tracer injection during coronary occlusion and before intervention (7, 25). Since this approach has been challenging to implement in clinical practice, CMR as a surrogate may enable AAR delineation and estimation of salvage. However, the limitation is that it seldom can delineate pre-interventional risk zones (29). This is one crucial question that remains in PICSO studies since the area at risk may be influenced by the intervention itself, which potentially cannot be depicted in *post hoc* measurements.

When we conceptualized PICSO, the first experimental study with 6 h of infarct without reperfusion resulted in an average of 45% reduction in infarct size related to AAR. An important observation was that the post-occlusive coronary artery pressure increased substantially, relating venous backflow to infarct size reduction and indicating the importance of collateralization. In 2004, Syeda et al. reported on a meta-analysis of different forms of retroperfusion showing the potential of PICSO, with a significant reduction of infarct size dependent on the achieved coronary sinus pressure during coronary sinus occlusion (30, 31). A statistically significant correlation between achieved coronary sinus pressure per minute (ratio between coronary sinus occlusion and release) and infarct size showed a negative association in the PICSO group (*r* = −0.92, *p* < .007). Recently, Egred et al. reported in patients with ACS treated with PICSO a significant absolute of 6.9%, a 33% relative decrease of infarct size as a percentage of left ventricular mass in a propensity-matched analysis compared to historical controls (INFUSE MI) measured by CMR 5 days after the infarct (26). This scientific evidence convincingly shows that the salvage potential of PICSO in the modern era of reperfusion interventions needs a conceptual upgrade to explore its full potential.

It became evident that PICSO's beneficial effects are related to collateral flow and the molecular and metabolic changes in the penumbra of infarcts. Hearse speculated about the existence of a border zone as a “salvageable myocardium.” Recently, Kung et al. characterized the zone using diffusion tension CMR, identifying regions of significant microstructural remodeling (32, 33). At the beginning of using coronary sinus interventions, the majority of opinions regarded the arterialized veins in the center of an infarct as the anatomical origin of salvage, disregarding that the effect started in border zones and the quality of the retroperfusate sampled in occluded coronary arteries was venous (6, 34, 35). Collateral flow and infarct size are firmly united and negatively related (36). Schopf et al. reported on the influence of PICSO on the regional nucleotide content and an increase of adenosine triphosphate (ATP) and adenosine diphosphate (ADP) in border zones (37). The improvement of regional myocardial function in severely depressed myocardium seems to be related to ATP levels found experimentally. Weigel et al. reported a PICSO-related increase of heme oxygenase (HO), a molecule known to inhibit post-myocardial infarct remodeling and restore ventricular function (18, 38). The vascular endothelial growth factor (VEGF) increased in the border zone of an infarct compared to controls, observing a statistically significant correlation (p < 0.01) between the coronary sinus pressure and the stretch-induced expression of HO-1 and VEGF mRNA, indicating that pressurization of the venous microcirculation is an effective stimulus in addition to ischemia for the manifold expression of these vasoactive molecules.

Even today, the target of mainstream PICSO therapy is to effectively access reperfused obstructed microcirculation and reduce the impedance of inflow, as shown by De Maria et al. in the Oxami trial (10).

A sudden influx of oxygenated blood and debris from a dilated and stented occlusion site into the reperfused myocardium as soon as reperfusion of a culprit lesion and infarct vessel occurs (39). Complex interdependencies of multiple cofounders add to what we know as reperfusion injury. Venous microcirculation as the primary zone of reperfusion injury turns venous access into deprived zones as a logical consequence. In 2012, Van de Hoef et al. reported on brief occlusions of coronary arteries with or without PICSO showing the successful duplicate entry into underperfused myocardium elevating post-occlusive pressures in coronary arteries in men as seen in experimental animals (25, 40).

Transcatheter coronary sinus interventions have been tested in different species. Most of the data acquired are from larger animals. Species-related anatomical differences in venous outflow play a significant role in the site of obstructing venous vasculature, as is the position of the balloon occlusion site. The amount of blood from normally perfused areas redirected to ischemic zones depends on the balloon blockade. The farther a catheter is advanced into the deprived zone, the smaller the amount of retroperfused blood. The retroperfused blood volume is reduced because of the lack of inflow of veins from the remote zones into the blocked cardiac veins. We found that the amount of retroperfused blood per minute depends on optimal occlusion release cycles and reaches about 30 ml per minute in experimental animals. No such measurements are reported in humans (27, 41).

Uncontrolled and permanent obstruction of venous drainage, primarily if it occurs acutely in awake patients, is dangerous since it limits arterial inflow (27, 34, 42). Therefore, studying regional and global hemodynamics is crucial during controlled PICSO application and is depicted in Table 2. “Acute effects on hemodynamics after PICSO in elective patients with reduced ejection undergoing resynchronization therapy revealed no significant change in global hemodynamics and transmyocardial metabolic parameters (43). Furthermore, none of these parameters show a differential pattern between a cohort of patients with chronic heart failure with and without 20 min of PICSO (43).”

**Table 2:** Global and coronary venous hemodynamic parameters in a heart failure cohort undergoing resynchronization therapy.

Hemodynamics			
	Control (*n* = 24)	PICSO (*n* = 8)	*p*-value
Cardiac index CI (L/min/m^2^)	2.58 ± 1.34	2.27 ± 0.72	0.4603
Stroke index SI (ml/m^2^)	32.8 ± 5.6	29.5 ± 9.2	0.2817
SVRI (dynes s/cm^5^/m^2^)	3,371 ± 1,739	3,043 ± 1,057	0.3039
MAP in mmHg	87.6 ± 14.1	81 ± 13.4	0.2312
HR in bpm	79.2 ± 6.8	77.7 ± 6.1	0.6391
PICSO hemodynamics			
	Control (*n* = 24)	PICSO (*n* = 8)	
Mean CSP pre-PICSO (mmHg)	—	11.73 ± 6.46	—
Mean CSP during PICSO (mmHg)	—	24.28 ± 7.47	—
Mean CSP post-PICSO (mmHg)	—	12.14 ± 5.7	—
Developed pressure (mmHgs^2^)	—	4,30,937.5 ± 1,19,507.45	—
Increase in mean CSP (%)	—	107%	—
Max CSP pre-PICSO (mmHg)	—	18.42 ± 10.20	—
Max CSP PICSO (mmHg)	—	52.18 ± 9.51	—
Max CSP post-PICSO (mmHg)	—	16.72 ± 5.93	—
MAP pre-PICSO (mmHg)	—	82.17 ± 12.09	—
MAP PICSO (mmHg)	—	80.01 ± 11.01	—
MAP post-PICSO (mmHg)	—	86.50 ± 16.22	—
Lactate extraction			
	Control (*n* = 24)	PICSO (*n* = 6)	*p*-value
Lactate extraction pre-OP (%)	27.67 ± 12.33	28.96 ± 10.21	0.5183
Lactate extraction intra-OP (%)	19.85 ± 14.57	15.07 ± 11.71	0.2109
Lactate extraction post-OP (%)	22.08 ± 12.30	20.63 ± 5.61	0.4367

MAP, mean arterial pressure; HR, heart rate; PICSO, pressure-controlled intermittent coronary sinus occlusion; CSP, coronary sinus pressure; ACS, acute coronary syndrome; CI, cardiac index; SI, stroke index, SVRI, systemic vascular resistence index; OP, operation.

Despite the significant increase in CSP, no differences can be seen in other circulatory parameters. Note that lactate extraction in elective patients after 20 min of PICSO is not influenced, as seen in post-MI patients in the Japanese study on PICSO in ACS (17), but improves significantly in the PICSO cohort after 24 h, see text (17).

This absence of PICSO effects in elective patients with chronic heart failure contrasts with patients with acute coronary syndromes, as reported from a historical Japanese study, showing beneficial results of PICSO on lactate extraction, reaching its highest difference 24 h after reperfusion (16).

In experimental animals and ongoing ischemia, coronary sinus systolic pressure increased over time, improving myocardial force. Monitoring coronary sinus pressure dynamics in PICSO patients in the early reperfusion period shows a decrease in the time to reach the systolic pressure plateau and a sudden decline as flow is restored, opening bypass grafts, indicating that flow increase in cardiac veins changes the pattern of systolic pressure increase (8).

## Branching points of research generate the hypothesis of “embryonic recall”

The current recognition of a mechanism of action of PICSO is based on two interdependent mechanisms. First, reopening the obstructed reperfused microcirculation and reducing intramyocardial resistance have been essential parameters in the chain of cardiac improvement. In this context, Scarsini et al. reported that the persistence of post-ischemic microvascular dysfunction predicts a more than four-fold increase in long-term risk of adverse outcomes, mainly driven by heart failure (10, 26, 44). The PICSO effects of filling-deprived microcirculation and changing resistance are dose–response dependent. The main denominator of success is the optimal amount of venous blood shifted from uncompromised cardiac zones backward and dependent on the site and effective balloon blockade of venous outflow (45).

Retrograde access to deprived myocardium and washout of toxic debris as evidence of PICSO has been accepted early (46). However, the data collected also sheds light on another essential feature of PICSO: activating vascular cells in cardiac veins initiates molecular signals, potentially triggering pathways known from cardiac organogenesis, leading to recovery (21).

There is still controversy about the therapeutic effect's primary mechanisms and the extent to which the mechanistic result of retroperfusion of venous blood into the deprived myocardial zones and microcirculatory dysfunction is supported by the activation of endothelium starting molecular activation via the mechanochemical signaling of PICSO.

Respecting the counterintuitive signs as the branching points from mainstream PICSO understanding was necessary, and we formulated a hypothesis, “Embryonic Recall.”

**Figure 2:**
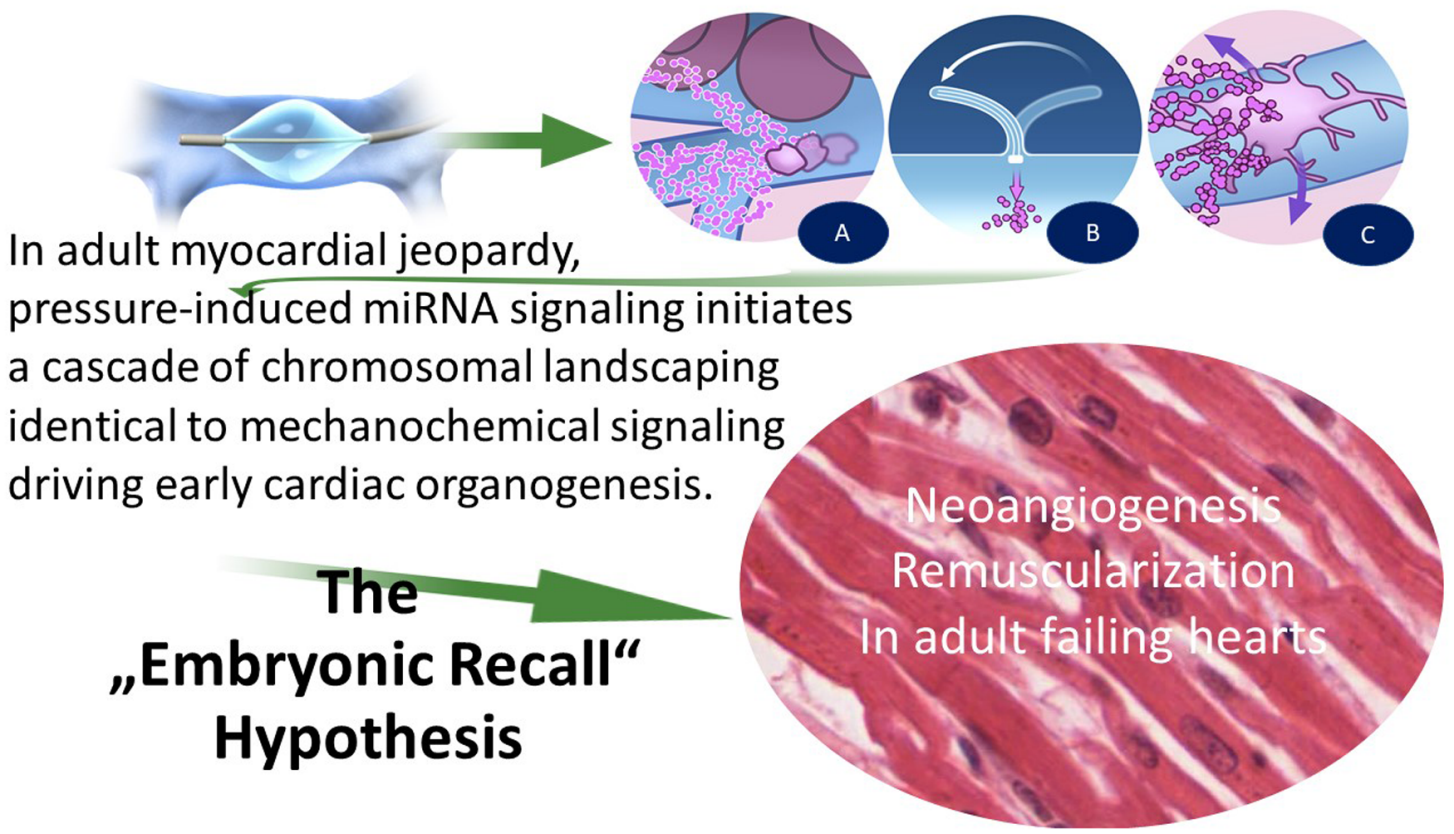
Treatment of microcirculation dysfunction and the initiation of developmental regenerative pathways using the mechanochemical feedback loop according to our hypothesis of “embryonic recall.” Mechanochemical process: PICSO uses the same canonical pathways as the early embryo shortly after the heart tube starts beating. Sensing this “mechanical event” triggers mechanochemical signaling progressing heart organogenesis. Likewise, PICSO uses the same “mechanical signal initiation” principles in the failing adult heart and influences via the same non-coding RNA (143/145 flow-sensitive miRNAs) epigenetic landscaping, building new vessels, and revascularizing failing hearts. Illustration of the principles of the “Embryonic Recall” hypothesis. During balloon occlusion, blood is forced into the microcirculation, and plasma is separated at the significant branches leaving a higher hematocrit in the coronary veins. Plasma enters the microcirculation. This “plasma skimming” process initiates the secretome-generating PICSO machinery. During the opening of the balloon occlusion, forward flow empties the microcirculation, and repetitive cycles induce washout. There are three hypothetical sources of molecular signals. (**A**) Separation of plasma and degranulation of platelets at branching points. (**B**) Venous cilia bend in the retrograde flow and activate the cytoskeleton inducing the mechanochemical feedback loop. (**C**) Pericytes are stretched by radial forces of retroflowing plasma. PICSO, pressure-controlled intermittent coronary sinus occlusion.

The central idea of our hypothesis, “Embryonic Recall,” and the molecular effects of PICSO are based on the similarities of mechanical influence on cardiac organogenesis and the theoretical revival in adult failing hearts. The principles of the hypothesis are depicted in Figure 2 and Table 3. A reversal of flow in the microcirculation of cardiac veins initiates a domino effect re-entering developmental pathways in adult failing hearts due to the activation of vascular cells. In short, the hypothesis proposes that the periodic increases of systolic pressure and retrograde flow into microcirculation and molecular signaling during cardiac development are based on identical mechanisms and can be revived in adult failing hearts using the mechanochemical feedback loop (20, 21, 50).

**Table 3:** Depicts claims and evidence supporting the “Embryonic Recall” hypothesis.

The “Embryonic Recall” hypothesis: analysis and verification	
The claim	Proposed rationalization
**Mechanotransduction regulates cardiac organogenesis in the developing heart**	
miRNA are critical regulators of correct cardiac development and are dependent on mechanochemical stimuli in embryos	Miyasaka 2011 described “hemodynamics-dependent processes of morphogenesis,” identifying “miRNA-143 as an essential component of the mechanotransduction cascade in zebrafish embryos (47). Vermot 2009 identified intracardiac reversal flow and shear-responsive genes in the developing heart linking intracardiac hemodynamic forces to valve development (48).
Yan 2016: non-coding RNA initiate angiogenetic, proliferative, and antiapoptotic molecules in cardiac development (49).	
**Mechanotransduction revived in the adult failing heart**	
Mohl 2007, 2014: formulation of the “embryonic recall” hypothesis. Hemodynamic force in cardiac veins initiates early embryonic pathways in cardiac organogenesis (50).	Activation and mechanotransduction of intermittent pressure increase in cilia of endothelial cells and stretching of pericytes, and degranulation of platelets, among presently other hitherto unknown sources, activate non-coding RNA.
PICSO, via mechanotransduction, activates miRNA and other unknown signaling initiating cardiac developmental pathways in the adult failing heart.	Mohl 2018: the first clinical evidence that PICSO-activated miRNA can be detected in cardiac venous blood. Compared with controls, there was significant differential miRNA expression in venous blood after 20 min of PICSO in patients with heart failure (51).
Shear stress-dependent miRNA activated by PICSO	Mohl 2022 shear-dependent miRNA 145-5p activated by PICSO in intact porcine cardiac tissue /see text)
PICSO and miRNA expression is related	Mohl 2022: significant relationships between pressure increase and miRNA levels are found in miRNA 19b and miRNA 101 (51). Weigel 2007 describes a significant correlation between developed peak coronary sinus pressure and the VEGF and HO-1gene transcription (18).
Elevation of pressure in veins is related to salvage and infarct size in experimental animals	Syeda 2004 establishes in a meta-analysis the correlation between reported pressures in cardiac veins and the reduction of infarct size (31).

PICSO, pressure-controlled intermittent coronary sinus occlusion.

The influence of endocardial activation as a driver in cardiac organogenesis started by mechanochemical feedback is well accepted in developmental research (52). This feedback occurs at a cellular level rearranging the cytoskeleton of endocardial and endothelial cells combining mechanical stimuli with biochemical signaling in development, leading to a cascade of canonical pathways forming the cardiac phenotype as we know it in adulthood.

Theoretically, and supported by new evidence, the same feedback between flow patterns and developmental pathways applies in the adult failing heart. Mechanotransduction associated with a cyclic pressure increase in cardiac veins through repetitive coronary sinus occlusion may stimulate this dormant embryonic machinery in adult hearts by activating the cytoskeleton of venous endothelium and pericytes. Multiplying this effect, the temporary redistribution of the cardiac microcirculatory flow eventually displaces molecules with paracrine effects into the myocardium by the pressure increase in cardiac veins (51).

In developing this hypothesis, we searched for a signaling molecule stable throughout the mammalian evolution and considered non-coding RNA the best-fitting potential target. In our first description of the molecular effects of PICSO, we focused on this signaling molecule. As a result, we were the first to describe the significant upregulation of the flow-sensitive miR-143/145 complex by PICSO in patients with heart failure, substantiating the core proposition of our hypothesis (43). Since we previously found cardiac venous blood-borne miRNA induced by PICSO, miR-145 was quantified in cardiac tissue (43).

In proving this hypothesis, we studied the flow-sensitive miR-143 and miR-145 complex expression as an indicator of cardioprotective signaling predicted by our theory in animals. In addition, to separate PICSO effects from changes in ischemic myocardium, we evaluated tissue samples of three anesthetized house swine, subjected to 4 h of PICSO in the intact heart in porcine myocardium in the venous flow and pressure-dependent left anterior descending (LAD) region as well as in the right ventricle (RV) with independent myocardial drainage.

The miR-145-5p was significantly 5-fold upregulated in both cardiac regions against the reference miR-21-5p (*p* < 0.05). Although miR-21 has repeatedly been associated with cardiac events such as response to oxidative stress, NF-kB activation, or fibrosis formation, its expression was chosen as an averagely expressed miRNA, which was subject to minor variation in our analyzed cardiac tissue samples, consistent with our theory, that PICSO alone does not change miR-21 expression (53). (Range of Ct values in qPCR: 25.6–26.8). Consequently, miR-21 was a suitable reference in our animal model, showing that miR-145-5p was abundantly expressed. Furthermore, its high expression is in line with its capacity to regulate smooth muscle cell fate and plasticity, protecting cardiomyocytes regulated by circular RNA (circ_0010729) with a profound effect on the regenerative capacity of smooth muscle cells after injury. The other strand of miR-145, i.e., miR-145-3p, remained low in the myocardial tissue, suggesting none or little influence by the PICSO intervention. Although miR145-3p is poorly described in the literature, it was recently advocated as a biomarker in a panel of miRNA, indicating acute myocardial infarction and poor clinical outcomes (54).

Miyasaka et al. found the same expression of the flow-dependent miRNA miR-143 as the outgoing molecular signal on the environmental/mechanical stimulus in organogenesis after heartbeat initiation in zebrafish. Furthermore, knocking down miR-143 results in the de-repression of retinoic acid signaling leading to abnormalities in ventricular outflow tracts, showing the importance of the signaling event in cardiac development (47).

The hypothesis proposes that hemodynamic-dependent morphogenesis processes can be revived in failing hearts by the same input signal as a regulator, in our case, activation of vascular cells by blood flow in cardiac veins, thus reopening the interdomain conjunction. Importantly, signaling is effective only when the specific environment, i.e., mechanical stimulus, is adequate, which we believe is caused by temporarily increasing the flow and pressure in cardiac veins.

## Changing the paradigm: PICSO, the secretome machine

As depicted in Figure 3, there are several sources of production signaling molecules by PICSO. First, a plasma skimming effect degranulates platelets, a significant source of miRNA, and forces plasma into the venous microcirculation leaving blood cells in more prominent veins; second, by bending cilia in endothelial cells; and third, by stretching pericytes, consequently leading to changes in the cytoskeleton of vascular cells and initiating the mechanochemical feedback loop. All three effects lead to the production of molecules, but which of these effects is the most powerful remains to be elucidated. After a myocardial injury, exosomes have recently been identified as an emergent therapeutic target in heart failure as potential anti-fibrotic agents. Although unproven, they are small vesicles packed with signaling molecules and may also be released by flow and pressure changes during PICSO (55).

Recently, Scarsini et al. published that PICSO in inferior STEMI is now the subject of another randomized trial (Clinicaltrials.gov NCT04958421) (56).

Since the posterior interventricular vein flow is typically unhindered by PICSO, we consider that activating collaterals and molecular events or soluble factors released and washed into the independent drainage zones are causing positive effects on vasodilation, as first described by Weigel et al. in 2007 (18).

## Changed signaling in patients with heart failure with PICSO

In 2018, we reported acute molecular changes in secreted molecules by PICSO. Eight out of 32 patients undergoing resynchronization device implantation were treated with PICSO for 20 min. Coronary sinus blood samples were collected before and after PICSO or 20 min in untreated patients, and pre-interventional sera were compared to post-interventional sera in both groups as well as among groups. The miRNA patterns between samples collected before and after the treatment and compared to controls were differentially expressed. In addition, these samples were used as a medium for cultures with cardiomyocytes from cardiac tissue harvested during heart transplantation, also displaying differences in proliferation (43). Compared with controls, we observed significant differential expression (differences between pre-values and post-values in both patient cohorts) of miRNA patterns with PICSO, generally associated with cardiac development.

Analyzing serum samples, comparing pre- and post-interventional sera and compared to controls, notably, miR-143 (*p* < 0.048) and miR-145 (*p* < 0.047) increased, both targeting a network of transcription factors, including KLF-4 (Krüppel-like factor 4), an evolutionarily conserved member of the KLF family of zinc finger transcription factors promoting differentiation and repress proliferation of vascular smooth muscle cells. In addition, other transcription factors showed favorable trends by PICSO, as MeF2a (myocyte enhancer factor 2a), a prominent member of the transcription family, acting as a myocardial enhancer in cardiac development and adaption to hypertension-induced cardiac hypertrophy, although not statistically significant, due to the small number of patient tested (57). Moreover, MeF2a has an essential role in cardiac development, and knockout results in embryonic lethality, defective chamber wall maturation, and reduced cardiomyocyte proliferation (58).

In addition, PICSO increased miR-19b (*p* < 0.019), alleviating endothelial cell apoptosis. The miR-17-92 cluster is a highly conserved gene cluster that encodes various conserved miRNAs, including miR-19b (59). Recently, Gao et al. described miR-19b in regeneration and protection from myocardial infarction by intracardiac injection, claiming that these miR-17-92 cluster members enhance cardiomyocyte proliferation (60). Promoting cardiomyocyte proliferation after injuries to reinitiate the process of cardiomyocyte regeneration and suppress heart failure is a potential target for treating heart failure (61). Using post-PICSO sera as a medium in cell cultures significantly increased cellular proliferation both in fibroblasts (*p* < 0.001) and adult cardiomyocytes (*p* < 0.004) harvested from a transplant recipient compared with controls. In addition, adult cardiomyocytes showed a seven-fold increase of the transcription factor KLF-4 protein when cultured with treated sera compared with controls. Increased miR-19b and the observed proliferation induced by PICSO suggest a causal relationship (43).

The significantly upregulated miR-19b and miR-101 values correlate to the time of pressure increase in cardiac veins during PICSO (*r*^2^ = 0.90, *p* < 0.05; *r*^2 ^= 0.98, *p* < 0.03), suggesting a flow- and pressure-dependent secretion of signaling molecules into the coronary circulation. Our observed relationship in patients with heart failure corroborates Weigel's correlation between heme oxygenase with the developed pressure in coronary veins during PICSO, indicating cytoprotection during ischemia/reperfusion injury. For the first time, this causal relationship connects the hemodynamic consequences of PICSO via signaling events to published evidence on the proliferation of cardiac cells (43, 61–64).

The second non-coding RNA showing a relationship with PICSO duration is miRNA-101. Recent research suggests that miR-101-attenuated myocardial infarction (MI)-induced injury by targeting DNA damage-inducible factor 4 (DDIT4) to regulate autophagy, indicating that miR-101 or DDIT4 may be potential therapeutic targets for heart injury and may play a role in apoptosis (62).

Interestingly, miR-101 plays a protective role against cardiac remodeling following MI via inactivation of the RUNX1 (runt-related transcription factor1), proposing miR-101 and RUNX1 as potential therapeutic targets for MI (64, 65).

Recently, Wang et al. showed that miR-101a-loaded mesenchymal stem cells in extracellular nanovesicles significantly improved heart function, decreased infarct size, and decreased fibrosis after MI even when injected into the myocardium 13 days after the infarct, suggesting the equivalent upregulation of miR-101 in PICSO patients as a favorable event (66).

Other disadvantageous non-coding RNA found in our study on acute changes in patients with heart failure influenced by PICSO are miR-320b and miR-25 (*p* < 0.023). Both miR-320b miR-25 (*p* < 0.023) decreased, and proposed targets of anti-miR application to improve contractility in the failing heart. Wang 2014 provided a novel mechanism for the exosomal miR-320 functionally downregulated its target genes IGF-1, Hsp20, and Ets2 (IGF-1, insulin-like growth factor; Hsp20, heat shock proteins acting in myocytes as chaperons; Ets2, belonging to the ETS family of transcription factors, is implicated in a broad range of cellular functions underlying diabetes mellitus-induced myocardial vascular deficiency caused by secretion of anti-angiogenic exosomes from cardiomyocytes) (67). In the same year, Wahlquist et al. reported an improvement in cardiac contractility inhibiting miR-25 (68).

Therefore, since signaling molecules sequestered into the coronary venous blood are differentially expressed by PICSO, and relate to pressure increase during PICSO, their role in ACS outcome can be predicted. Relating PICSO parameters to PICSO effects on non-coding RNA coincide with the report by Syeda et al., convincingly showing that the sequence of occlusion release phases and the increase in systolic pressure over time is related to the amount of myocardium salvaged (31). Although our retrospective pilot study is only an indication, because of the small number of patients tested and need to be tested in pivotal studies, it is a first glance at the advent of PICSO in the potential to treat myocardial jeopardy in acute coronary syndromes and heart failure beyond the noticeable perfusion changes.

Also, the contribution by Weigel et al. was found in a positive and statistically significant correlation between mean and developed peak coronary sinus pressure and the HO-1 and VEGF gene transcription. These findings are now completed with a relationship between signaling molecules and PICSO parameters, strongly suggesting that mechanical strain and stretch produced by the pulsatile action of the reflowing blood in the deprived venous vasculature triggers the activation of a genomic program within the myocardium (18).

## The PICSO paradigm in the context

Clearing ischemic/reperfused microcirculation and its relationship to myocardial salvage is the mainstream PICSO claim to be tested in ongoing clinical trials. PICSO can influence the pathophysiology of structural changes in the post-reperfusion myocardium. The plasma skimming theorem of PICSO was tested using microhematocrit changes in cardiac veins substantiating measurements of blood density as the crucial parameter of PICSO effectiveness. Separating cellular components in more prominent veins and pressing plasma into the microcirculation, thus varying hematocrit levels measured in the coronary sinus, is the typical denominator improving conductance in the reperfused microcirculation. This effect seems to be the principal cause of impact on molecular secretion (see Figure 3). A more extended duration of PICSO induces a trend toward lower density measurements (see Figure 1). It can be interpreted as a washout, as described by Kenner and Moser in their seminal analysis, showing a close relationship between systolic pressure in cardiac veins after 20 min of PICSO and a reduction of the blood density gradient between the aorta and coronary sinus, indicating washout (9, 69). These experimental findings and the clinical reports from De Maria et al. on the microcirculatory resistance index must be seen in context to the clinical outcome as analyzed by de Waha et al. reporting on the time course to new cardiac events (10, 70).

In our long-term observations after the first clinical “Japanese” PICSO study in patients with AMI, the incidence of stenosis was about 8% less after PICSO compared with the control group (*p* < 0.044) (16).

Furthermore, a strong relationship between restenosis and MACE was observed (*p* < 0.0149). Adjusting for restenosis, the risk for MACE was nine times higher for the control group than for the PICSO group (16).

This observation is in context with a recent meta-analysis, which stated that abnormal coronary flow reserve (CFR), as an index for injured microcirculation, was also associated with a higher incidence of MACE in patients with acute coronary syndromes (71). Furthermore, correcting washout, now extensively proven as one of the dual principles of PICSO, is a phenomenon reestablishing homeostasis of puffer systems and inducing vasodilation in the coronary circulation, which may also influence outcome after PICSO (10, 15, 19, 21). In addition, washing out plugged thrombi from the venous microcirculation has been reported previously and may play a significant role in this process (72).

The second important mechanism, interpreting our results on restenosis and MACE, is the molecular signaling event, influencing the relationship between intrinsic apoptosis, necroptosis, and pyroptosis, influencing infarct healing and correcting the inflammatory process in ACS. In our study, the recurrence of reinfarction was reduced. The relative risk for re-MI was 17 times higher for the control group than for the PICSO group, even after including other risk factors (16).

In a recent article, Crea summarized the importance of inflammation in post-ischemic events, implying the inflammatory risk and its relationship to the recurrence of ischemic events (73).

As said, PICSO seems to activate alternate pathways limiting inflammation and reopening canonical pathways described in our “Embryonic Recall” hypothesis. In addition, the plasma skimming machinery of PICSO next to producing molecular signals reopens the venous microcirculation, liberating edema and toxic metabolites.

Molecular events during reperfusion of a deprived myocardial territory are a complex problem with multiple signaling and sometimes counteracting events. Therefore, it can be expected that a burst of several non-coding RNA, many hitherto unknown, in context with other regenerative molecules, are involved in the signaling event, both induced by the disease process and by PICSO (43).

Reversal of adverse remodeling to prevent heart failure is one of the targets of recent research, and measurements of ventricular volumes are a diagnostic option (74, 75). In this context, it is noteworthy that in the analysis by Egred et al., left ventricular enddiastolic volume (LVEDV), measured by CMR 5 days after the infarct in patients treated with PICSO, showed a significantly smaller volume than controls, indicating a potential benefit in the remodeling process (26). The knowledge of the complex interconnected network of non-coding RNA as signal mediators and the role of PICSO mechanically inducing the signaling event is essential in understanding PICSO and its potential to change the course of necroptosis, pyroptosis, and apoptosis after reperfusion of infarcts preventing adverse remodeling and heart failure.

## Paradigms of signs and signals dictate rewriting the PICSO perspective

We are expecting results of ongoing clinical trials of PICSO research since they will eventually answer the questions asked 30 years ago and whether it is safely applicable in the routine logistics of cath labs in the 2020s, correcting reperfusion injury. It remains to be seen whether infarct size reduction is the correct signal, relating patient outcome with apparent advantages for patient survival and quality of life.

These hypothesis-generating observations of a noticeable paradigm change will guide the exploration of the novel approach to treating myocardial microcirculation during jeopardy. In addition to salvage, an overall cardioprotective effect can be anticipated, leveraging problematic infarct size measurements as expected in our hypothesis. Hemodynamic PICSO forces reopen the ischemic/reperfused microcirculation, mainly by redistribution of flow and washout, but initiating cascades of molecular signaling seems to be an essential factor as well. In the next step, corroborating molecular signs and signals of PICSO effects in pivotal trials is mandatory since we must verify heart failure studies of non-coding RNA in patients with acute myocardial infarction. More extensive, primarily clinical studies will eventually confirm molecular signaling as one of the significant effects and establish PICSO as valuable therapy for ischemic, reperfused microcirculation treatment in patients with STEMI and beyond.

The future of clinical PICSO is based on an analytical appraisal of yesterday's conceptualization, an open eye for identifying cross roads, even unexpected counterintuitive findings, and an available vision of the future's scientific perspective in parallel to clinical evidence.

This notion and the uncertainty of whether an additional infarct size reduction can be achieved as an add-on therapy during reperfusion dictates another paradigm change in PICSO research, concentrating on repair pathways and starting even before reperfusion. This newly defined PICSO target needs sophisticated technology and new clinical research perspectives to mitigate patient outcomes.

## Limitations

The information presented here is a roadmap of findings with counterintuitive results forcing research into unexpected territories. What started as an anti-ischemic procedure developed into a complex signaling and network of molecular pathways. Abstracting scientific evidence warranted the formulation of our hypothesis, “embryonic recall.” It is apparent, however, that tested miRNAs are not the only signaling pathways, and many more molecular signals must be detected.

The development of the PICSO concept bridged several decades and was started before revascularization therapies became routine. The current understanding of “time is muscle” and early revascularization nowadays limiting PICSO applications into the reperfusion period falls short, considering ambivalent results in determining reperfusion injury.

Another significant limitation is that the administration of heparin interacts with miRNA activity as routine treatment in acute coronary syndromes and may be a serious disadvantage of a PICSO application.

Therefore, future research must concentrate on the molecular role of PICSO in routine revascularization procedures. It may very well be that an application during ischemia on the way to the cath lab or at any other time during infarct healing supports recovery and may better interact with myocardial decay.

## Data Availability

The original contributions presented in the study are included in the article, further inquiries can be directed to the corresponding author.
